# When Just One Sense Is Available, Multisensory Experience Fills in the Blanks

**DOI:** 10.1371/journal.pbio.0040361

**Published:** 2006-09-26

**Authors:** Liza Gross

Our brains are wired in such a way that we can recognize a friend or loved one almost as easily whether we hear their voice or see their face. Specialized areas of the brain—in this case, the visual and auditory networks—are specially tuned to different properties of physical objects. These properties can be represented by multiple sensory modalities, so that a voice conveys nearly as much information about a person’s identity as a face. This redundancy allows rapid, automatic recognition of multimodal stimuli. It may also underlie “unimodal” perception—hearing a voice on the phone, for example—by automatically reproducing cues that are usually provided by other senses. In this view, as you listen to the caller’s voice, you imagine their face to try to identify the speaker. In a new study, Katharina von Kriegstein and Anne-Lise Giraud used functional magnetic resonance imaging (fMRI) to explore this possibility and understand how multimodal features like voices and faces are integrated in the human brain.

Studies using fMRI have established that when someone hears a familiar person’s voice, an area of the temporal lobe called the fusiform face area (FFA) is activated through temporal voice areas (TVAs), suggesting early interactions between these cortical sensory areas. von Kriegstein and Giraud wondered whether these cortical ensembles might lay the foundation for general “multisensory representation” templates that enhance unimodal voice recognition.

To explore this question, the researchers analyzed changes in brain activity and behavior after people learned to associate voices with an unfamiliar speaker. One group of participants learned to associate voices with faces and a second group learned to associate voices with names. Though both types of learning involve multimodal associations, voices and faces provide redundant information about a person’s identity (such as gender and age), while voices and names provide arbitrary relationships (since any name could be associated with any voice). To further explore the contribution of redundant stimuli from the same source, the researchers added an additional set of conditions in which participants associated cellular phones with either ringtones or brand names. In this case, both cell phone and brand name were arbitrarily associated with a ringtone.

In the first phase of the fMRI experiment, participants listened to and identified either voices or ringtones. In the second phase, one group of participants learned to associate the voices and ringtones with faces and cell phones, while another group learned voice–name and ringtone–brand name associations. In the third phase, participants again heard only the auditory signals and identified either voices or ringtones as in phase one.

**Figure:**
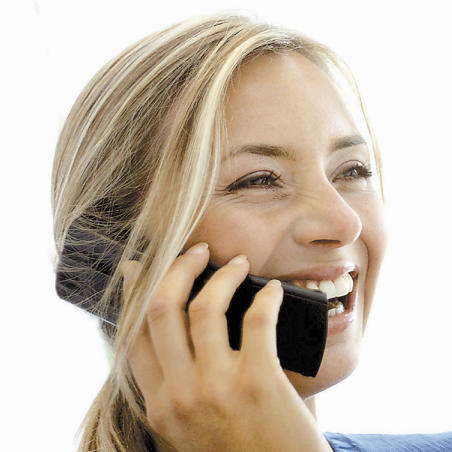
Recognizing people on the phone: Does knowing the face help? (Photo: copyright FEEI/FMK)

The brain scans showed that learning multisensory associations enhanced those brain regions involved in subsequent unimodal processing for both voice–face and voice–name association. But at the behavioral level, participants could recognize voices that they had paired with faces much more easily than they could recognize voices they had linked to names. Participants who had learned to associate voices with faces were the only ones to show increased FFA activation during unimodal voice recognition.

The fMRI results show that even a brief association between voices and faces is enough to enhance functional connectivity between the TVA and FFA, which interact when a person recognizes a familiar voice. In contrast, voice–name association did not increase interactions between voice and written name sensory regions. Similarly, people did not recognize ringtones any better whether they had learned to associate them with cell phones or brand names. Nor did their brain scans reveal any interactions between auditory and visual areas during ringtone recognition.

Altogether, these results show that learning voice–face associations generates a multimodal sensory representation that involves increased functional connectivity between auditory (TVA) and visual (FFA) regions in the brain and improves unimodal voice recognition performance. When only one sensory modality of a stimulus is available, the researchers conclude, one can optimally identify a natural object by automatically tapping into multisensory representations in the brain—cross-modal ensembles that are normally coactivated—as long as the stimulus provides redundant information about the object. Given that faces and voices are the major means of social communication for nonhuman primates as well as for humans, the reliance of multiple, redundant sensory modalities likely has deep roots in our evolutionary history.

